# Practical diets for California yellowtail, *Seriola dorsalis*: Use of advanced soybean meal products on growth performance, body composition, intestinal morphology, and immune gene expression

**DOI:** 10.1371/journal.pone.0304679

**Published:** 2024-06-07

**Authors:** Abdulmalik A. Oladipupo, Kevin R. Stuart, Timothy J. Bruce, Mark A. Drawbridge, D. Allen Davis

**Affiliations:** 1 Auburn University, School of Fisheries, Aquaculture and Aquatic Sciences, Auburn, Alabama, United States of America; 2 Hubbs-SeaWorld Research Institute, San Diego, California, United States of America; Tanta University Faculty of Agriculture, EGYPT

## Abstract

California yellowtail (CYT), Seriola dorsalis, is a promising candidate for aquaculture due to its rapid growth and high-quality flesh, particularly in markets like Japan, Australia, China, and the United States. Soy protein has shown success as a replacement for marine protein sources in CYT diets, reducing fishmeal levels, though concerns about potential intestinal inflammation persist with the inclusion of solvent-extracted soybean meal. To address this, processing strategies like fractionation, enzymatic treatment, heat treatment, and microbial fermentation have been employed to mitigate the negative impacts of soybean meal on fish nutrition and immune systems. This study focuses on optimizing soybean meal inclusion levels by incorporating advanced soy variants into CYT diets. The eight-week feeding trial, conducted in a recirculation system, featured six diets with sequential inclusion levels (0, 50, 100%) of high protein low oligosaccharide soybean meal (Bright Day, Benson Hill, St Louis, MO) and enzyme-treated soybean meal (HP 300, Hamlet Protein Inc., Findlay, OH), replacing solvent-extracted soybean. The study compares these formulations against a soy-free animal protein-based diet. At the end of the trial, fish were sampled for growth performance, body proximate composition, intestinal morphology, and immune response from gut samples. Results showed consistent FCR (*P* = 0.775), weight gain (*P* = 0.242), and high survival rate (99.4 ± 0.5%) among dietary treatments (*P*>0.05). Histological evaluations revealed no gut inflammation and gene expression analysis demonstrated no significant variations in immune, physiological, and digestive markers *apn* (*P* = 0.687), *mga* (*P* = 0.397), *gpx1* (P = 0.279), *atpase* (P = 0.590), *il1β* (*P* = 0.659). The study concludes that incorporating advanced soybean meal products, replacing up to 20% of fishmeal does not negatively affect CYT’s growth and intestinal health. This suggests that all three soy sources, contributing 35% of total protein (15.4 g 100 g^-1^ diet), can be included in practical diets without compromising CYT’s intestinal integrity or growth. These findings have positive implications for the commercial production of CYT and future research on the incorporation of plant-based proteins in aquaculture diets.

## Introduction

The use of plant proteins in fish feed has become imperative due to the steady increase in global fish feed production and the need to reduce the use of fishmeal in feed formulations. Among the plant-based proteins, solvent-extracted soybean meal has emerged as a prominent protein source in fish feed, attributed to soybeans high yield, widespread availability, and protein content [1, 2]. Nonetheless, investigations have revealed that incorporating soybean meal as a protein source can potentially affect growth performance, compromise immune functions, and heighten susceptibility to stress in certain aquatic organisms [3–5]. This is attributed to the presence of anti-nutritional factors (ANFs) i.e., trypsin inhibitors, saponins, glycine, and phytate, which can detrimentally impact feed digestibility, induce alterations in intestinal morphology, contribute to enteritis, and impede the activities of intestinal digestive enzymes in some species [6–8].

Numerous studies have explored the nutritional and immunological prospects of improved soybean derivative products obtained through enzymatic actions [9, 10], heat treatment [11], or microbial fermentation [12–14]. These strategies have been suggested to reduce the impact of ANFs and improve the nutritional values of soybean as a plant protein source in fish diets [10, 15], making these soy products commercially available for aquaculture. Fermented soybean products have been reported to improve growth performance and biological activities when included in the diets of shrimp (*Litopenaeus vannamei*) [16], Nile tilapia (*Oreochromis niloticus*) [17], coho salmon (*Oncorhynchus kisutch*) [18] and rainbow trout (*Oncorhynchus mykiss*) [19, 20] as a partial replacement of fishmeal. Enzyme-treated soybean meal could reduce the alteration in intestinal morphology in fish, thus leading to improved feed intake and growth performance [21]. Although Kumar et al. [21] discerned this improvement in rainbow trout, another enzyme-treated soybean product (Hamlet Protein 300) did positively influence a healthy gut, but growth performance was indifferent [22]. While this variability exists, processing techniques have contributed to improved digestibility of soybean products in fish species.

The California yellowtail (*Seriola dorsalis*; CYT), previously classified under the scientific name *Seriola lalandi*, is renowned for its rapid growth, desirable texture, and flavor. Its acceptance is reflected in the extensive commercial cultivation of other *Seriola* species in Japan, Australia, and New Zealand, where *Seriola* holds a significant place in creating diverse country-wide delicacies [23, 24]. Furthermore, CYT has successfully carved out a robust market presence in the United States, making it an attractive candidate for aquaculture production [25]. The practical diet of CYT has been reported to include 40–45% protein content [26] including soybean meal content of up to 30% [2]. CYT’s high tolerance to soy derivatives has been associated with improved growth performance, survival rates, feed conversion efficiency, and absence of enteritis [2, 26]. However, Viana et al. [27] found intestinal inflammation associated with fishmeal replacement with varying levels of soybean meal in CYT’s diet. This study aimed to further improve soy-based practical diets by assessing the effects of substituting standard solvent-extracted soybean meal with different soy derivatives on the growth performance, body composition, intestinal morphology, and immunity of CYT. The study incorporated fermented soy products and a genetically improved soybean meal variant, juxtaposed with commercial soybean meal at varying inclusion levels, and was compared against a soy-free animal protein-based diet, which included 30% fishmeal and 20% poultry meal as the primary protein sources.

## Materials and methods

### Ethical statement

The authors cite compliance with the US National Research Council’s ‘Guide for the Care and Use of Laboratory Animals’, the US Public Health Service’s ‘Policy on Humane Care and Use of Laboratory Animals’, and ‘Guide for the Care and Use of Laboratory Animals’. The Institutional Animal Care and Use Committee (IACUC) protocol used for this study was 2022–01.

### Experimental diets

Diets were produced at the E.W. Shell Fisheries Center of Auburn University, AL, USA. Pre-ground dry ingredients were mixed with menhaden fish oil in a food mixer (Hobart, Troy, OH, USA) for 15 min. Boiling water was then blended into the mixture to attain a consistency appropriate for pelleting. The moist mash from each diet was passed through a die (2 or 3 mm) in a meat grinder, and the pellets were dried in a forced air-drying oven (<50 °C) to a moisture content of less than 10%. Diets were stored at −20 °C and sieved to an appropriate size before use. Diets were analyzed for proximate and amino acid composition at the University of Missouri Agricultural Experiment Station Chemical Laboratories (ESCL; Columbia, MO, USA) (Table 1). All analytical methods complied with the Association of Official Analytical Chemists (AOAC) standards.

**Table 1:** Proximate composition of six experimental diets offered to California yellowtail (*Seriola dorsalis*) for an eight-week trial (as is, g 100g^-1^). Basal = 45% commercial SBM control diet; BD50 = 45% commercial SBM + enzyme-treated SBM diet; BD100 = 45% enzyme-treated SBM only diet; HP50 = 45% commercial SBM + fermented SBM diet; HP100 = 45% fermented SBM only diet; Reference = 45% fishmeal control.

	Treatments					
Proximate composition (g 100g^-1^)	Basal	BD50	BD100	HP50	HP100	Reference
Crude protein	45.79	46.54	44.88	46.84	43.85	44.43
Moisture	7.17	6.34	9.38	5.1	10.49	9.39
Crude Fat	9.62	9.56	9.21	9.64	8.81	8.73
Crude Fiber	2.25	2.02	1.94	2.21	2.42	1.24
Ash	8.59	8.57	8.09	8.62	8.03	8.64
Phosphorus	1.46	1.49	1.38	1.59	1.38	1.41
Taurine §	0.8	0.81	0.79	0.79	0.77	0.97
Hydroxyproline	0.45	0.48	0.44	0.52	0.46	0.69
Aspartic Acid	3.91	3.91	3.79	3.88	3.78	3.32
Threonine	1.65	1.65	1.59	1.65	1.61	1.57
Serine	1.79	1.78	1.74	1.81	1.74	1.58
Glutamic Acid	7.68	7.73	7.55	7.76	7.32	6.5
Proline	3.02	2.99	2.88	3.01	2.82	2.75
Lanthionine §	0.05	0.06	0.06	0.06	0.06	0.04
Glycine	2.52	2.59	2.46	2.62	2.48	2.94
Alanine	2.59	2.61	2.5	2.63	2.46	2.7
Cysteine	0.63	0.61	0.57	0.6	0.58	0.5
Valine	2.16	2.15	2.07	2.17	2.06	1.98
Methionine	1.01	1	0.96	1.03	0.97	0.98
Isoleucine	1.99	1.97	1.91	1.99	1.89	1.73
Leucine	3.95	3.95	3.84	4	3.74	3.55
Tyrosine	1.64	1.64	1.59	1.64	1.57	1.39
Phenylalanine	2.17	2.16	2.12	2.2	2.08	1.85
Hydroxylysine	0.06	0.06	0.06	0.06	0.06	0.09
Ornithine §	0.03	0.04	0.03	0.04	0.04	0.04
Lysine	2.47	2.45	2.37	2.43	2.3	2.49
Histidine	1.05	1.05	1.02	1.05	0.99	0.97
Arginine	2.67	2.71	2.63	2.71	2.61	2.39
Tryptophan	0.45	0.44	0.42	0.44	0.42	0.38
Total	44.74	44.84	43.39	45.09	42.81	41.4

Six diets were formulated on isonitrogenous and isolipidic basis for this trial (Table 2). The primary animal protein sources in all the diets were menhaden fishmeal and poultry meal. Soybean meal products as plant protein include solvent-extracted (SE) soybean meal (SBM), enzymatically treated SBM containing (HP300, Hamlet Protein, Finlay, OH), and a high protein low oligosaccharide SBM variety (Bright Day, St Louis, MO). HP-300 is a soy-based product derived from defatted soybeans through a unique proprietary process [28, 29]. As the product description specifies, this process reduces soybean oligosaccharides, trypsin inhibitors, lectins, and antigens. Bright Day SBM is also a low oligosaccharide higher protein soybean variety for which the meal is produced using traditional solvent extraction technologies. The basal diet was designed to contain 30% solvent-extracted soybean meal. This was sequentially replaced (0, 50, 100%) with Bright Day or HP300 at an equal protein basis. The sixth diet was formulated to contain 30% fishmeal, 23.8% poultry meal, and 8% corn protein concentrate, resulting in a soybean meal-free feed formulation.

**Table 2:** Experimental diet composition of the six formulated diets used in the feeding trials. Ingredient values are presented on a dry matter basis. Basal = 45% commercial SBM control diet; BD50 = 45% commercial SBM + enzyme-treated SBM diet; BD100 = 45% enzyme-treated SBM only diet; HP50 = 45% commercial SBM + fermented SBM diet; HP100 = 45% fermented SBM only diet; Reference = 45% fishmeal control.

	Basal	BD50	BD100	HP50	HP100	Reference
Menhaden fishmeal ^1^	100.0	100.0	100.0	100.0	100.0	300.0
Poultry meal ^2^	200.0	200.0	200.0	200.0	200.0	238.0
SE Soybean meal ^3^	344.0	172.0		172.0		
SBM Bright Day ^4^		144.0	289.0			
SBM HP 300 ^5^				139.0	279.0	
Corn protein concentrate ^6^	80.0	80.0	80.0	80.0	80.0	80.0
Menhaden fish oil ^7^	60.7	61.1	61.6	59.3	57.8	38.7
Corn Starch ^8^	0.4	26.0	52.5	32.8	66.3	152.8
Whole wheat ^9^	173.0	175.0	175.0	175.0	175.0	175.0
Mineral premix ^10^	2.5	2.5	2.5	2.5	2.5	2.5
Vitamin premix ^11^	5.0	5.0	5.0	5.0	5.0	5.0
Choline chloride ^8^	2.0	2.0	2.0	2.0	2.0	2.0
Rovimix Stay-C 35% ^12^	1.0	1.0	1.0	1.0	1.0	1.0
CaP-dibasic ^8^	25.0	25.0	25.0	25.0	25.0	0.0
Methionine ^13^	1.4	1.4	1.4	1.4	1.4	0.0
Taurine ^13^	5.0	5.0	5.0	5.0	5.0	5.0

^1^ Special Select^™^, Omega Protein Inc., Houston, Texas. 64% protein

^2^ River Valley Ingredients., 1170 Country Road 508. PO. Box 429 Hanceville, AL. 67% protein

^3^ Dehulled Solvent Extracted Soybean Meal, Bunge Limited, Decatur, AL. 45% protein,.

^4^ Benson Hill, St Louis, MO., 55.1% protein.

^5^ Hamlet Protein Inc., Findlay, OH., 56.0% protein.

^6^ Empyreal 75^™^, Cargill Corn Milling, Cargill Inc., Blair, Nebraska. 75% protein.

^7^ Omega Protein Inc., Reedville, Virginia, USA.

^8^ MP Biomedicals Inc., Solon, OH, USA.

^9^ Bobs Red Mill Natural Foods, Milwaukie, OR, USA.

^10^ Trace mineral premix (g/100g premix): cobalt chloride 0.004, cupric sulphate pentahydrate 0.250, ferrous sulphate 4.0, magnesium sulphate anhydrous 13.862, monohydrate 0.650, potassium iodide 0.067, sodium selenite 0.010, zinc sulphate heptahydrate 13.193, filler 67.964.

^11^ Vitamin premix (g/kg premix): Thiamin HCl0.751, riboflavin4.505, pyridoxineHCl1.502, D-Pantothenic acid hemicalcium salt7.508, nicotinic acid 7.508, biotin 0.075, folic acid 0.270, vitamin B12 0.003, inositol 7.508, menadione 3.003, vitamin A acetate (500,000 IU/g) 0.300, vitamin D3 (1,000,000 U/g) 0.60, DL-α-tocopheryl acetate (250/ IU g-) 12.012, α-cellulose 804.847.

^12^ Stay C^®^, (L-ascorbyl-2-polyphosphate 35% Active C), Roche Vitamins Inc., Parsippany, New Jersey, USA.

^13^ TCI (Tokyo Chemical Industry), Portland, OR, USA.

### Fish and feeding trial

The feeding trial was conducted in a twenty-four-tank (320L each) recirculation system at HSWRI’s research laboratory in San Diego, CA. CYT fingerlings were sourced from broodstock held at HSWRI and acclimated for 48 h in trial tanks. At the start of this trial, 15 CYTs of similar size (16.52 ± 0.01 g) were stocked into the tanks, which were subsequently randomly assigned to the six dietary treatments in 4 replicates. The fish were hand-fed to apparent satiation twice daily (08:00 am and 04:00 pm) for eight weeks, and feed intake was recorded daily. Ammonia (0.14±0.02 mg L^-1^), nitrite (0.03±0.00 mg L^-1^), nitrate (0.96±0.09 mg L^-1^), salinity (35.0±0.0 mg L^-1^), and pH (7.72±0.03) were monitored weekly, while dissolved oxygen (9.15 ± 0.07 mg L^-1^) and temperature (21.01± 0.20°C) were monitored daily. Temperature, dissolved oxygen, and pH were measured with a model HQ40d meter (Hach Company, Loveland, CO, USA). Total ammonia, nitrite, and nitrate were measured with a model DI/890 colorimeter (Hach Company, Loveland, CO, USA).

### Sampling and analysis

Fish were counted and batch-weighed at the start of the study and the end of the feeding trial. Fish were not anesthetized during the batch weights and were euthanized with a lethal dose of buffered MS-222 (250 mg L^-1^; Syndel Inc., Ferndale, WA, USA) at the termination of the trial. Diets and whole-body fish samples were analyzed for proximate composition (PC) analysis by Midwest Laboratories, Inc. (Omaha, NE, USA). All analytical methods complied with the Association of Official Agricultural Chemists (AOAC). Diet samples were taken at the start of the trial. Fish samples were taken at the beginning and end of the trial. For the initial sampling, 20 whole fish were euthanized in buffered MS-222 and then frozen at −80°C. Three whole fish were collected from each replicate tank for the final fish sampling, euthanized, and then frozen at −80°C. At the end of the trial, gross necropsies were performed on three fish per replicate tank (n = 72) to collect 2 cm x 2 cm sections of the distal intestine for histopathologic evaluation. Another 3-cm piece of the posterior intestine was dissected and immediately immersed in DNA/RNA Shield (Zymo Research Corp., Irvine, CA, USA) for 12 h at 4°C and then preserved at −20°C for gene expression. Survival (%), feed conversion ratio (FCR), and percent weight gain (WG %) were calculated as follows:

$$Percent\;weight\;gain\left(WG,\%\right)=100*\left[final\;body\;weight\left(g\right)-initial\;body\;weight\left(g\right)\right]/initial\;body\;weight\left(g\right)$$

$$Feed\;conversion\;ratio\;\left(FCR\right)=feed\;intake\;\left(g\right)/\left[final\;body\;weight\;\left(g\right)-initial\;body\;weight\left(g\right)\right]$$

$$Protein\;retention\;efficiency\left(PER\right):\left[\left(final\;total\;body\;protein-initial\;total\;body\;protein\right)/total\;dietary\;protein\;intake\right]\times 100.$$

$$Survival\left(\%\right)=100*\left[final\;quantity/initial\;quantity\right]$$

### Intestinal histology

Portions of the distal intestine were preserved in Bouin’s fixative for 24 h and then transferred to 70% ethanol. Preserved tissues were sent to the Auburn University Scott-Ritchey Research Center (Auburn University, Auburn, AL, USA) where they were timed, dehydrated, embedded in paraffin sectioned at 5 μm and stained with hematoxylin and eosin. A total of 72 slides were processed, three fish per tank (12 from each dietary treatment). Slides were examined using 100x magnification (Nikon E200, Melville, NY, USA). Segments were evaluated using a previously reported methodology (S1 Fig; Table 3) [19]. Three separate reviewers independently analyzed all slides at random, and ranking was performed on each slide to differentiate histopathologic changes in the intestine among diets based on the overall intestinal appearance and composition. Criteria assessed included the lamina propria, the amount of connective tissues beneath the mucosal folds, and the relative amount of large vacuoles present in the folds. Assessed ranks were compiled and averaged for overall gut scoring.

**Table 3:** Histological scoring system used on California yellowtail fed different variants of soybean meal diets (modified from Barnes et al. [19]).

Score	Appearance
	**Lamina propria of simple folds**
1	Thin and delicate core of connective tissue in all simple folds
2	Lamina propria is slightly more distinct and robust in some of the folds
3	Clear increase in lamina propria in most of the simple folds
4	Thick lamina propria in many folds
	**Connective tissue between the base of folds and stratum compactum**
1	Very thin layer of connective tissue between base of folds and stratum compactum
2	Slightly increased amount of connective tissue beneath some of the mucosal folds
3	Clear increase of connective tissue beneath most of the mucosal folds
4	Thick layer of connective tissue beneath many folds
	**Vacuoles**
1	Large vacuoles are absent
2	Very few large vacuoles are present
3	Large vacuoles are numerous
4	Large vacuoles are abundant in present in most epithelial cells

### RNA extraction, cDNA synthesis, and quantitative RT-PCR expression

RNA was extracted from the distal intestine of CYT using the Quick-RNA MiniPrep Plus kit (Zymo Research Corp., Irvine, CA, USA) according to the manufacturer’s recommendations. After the extraction, the concentration and quality of the RNA were confirmed with a Nanodrop One^c^ spectrophotometer (ThermoFisher Scientific, Waltham, MA, USA), ensuring the 260/280 ratios were between 1.8 and 2.0. Extracted RNA samples were diluted to 20 ng μL^-1^ according to the manufacturer’s instructions, and RNA was synthesized into cDNA using a High-Capacity cDNA Reverse Transcription Kit (Applied Biosystems, Waltham, MA, USA). Briefly, each 20 μL reaction contained 2 μL of 10x RT buffer, 0.8 μL of 25x dNTP Mix, 2 μL of 10x RT random primers, 1 μL of kit Multiscribe reverse transcriptase, 10 μL of template RNA and 4.2 μL of nuclease-free water. cDNA samples were synthesized in a Mastercycler X50s (Eppendorf, Enfield, CT, USA). Program conditions were set at 25°C for 10 minutes, 37°C for 120 minutes, and 85°C for 5 minutes. After the reaction, the cDNA was stored at -20°C. Quantitative real-time PCR was conducted with QuantStudio 5 Real-Time PCR (Applied Biosystems) using the PowerUp SYBR Green Master Mix (Applied Biosystems). Five genes of interest, *apn* (aminopeptidase N), *atpase* (sodium/potassium transporter), *gpx-1*(glutathione peroxidase -1), *mga* (maltase-glucoamylase), and *il1β* (interleukin-1β) were assessed, and the relative quantification of each gene (i.e., ΔCt) was determined through normalization against a housekeeping gene (beta actin; *actb)*. PCR primers used in this study (previously published by Viana et al. [27]) are described in Table 4. Prior to the start of the assays, primer efficiencies were assessed at an annealing temperature of 60 °C, primer concentration (500 nM), and template concentration (five 1:10 dilution series from 10 ng to 100 ng of input RNA). All cDNA samples were diluted to 1.25 ng μL^-1^ prior to the reaction. Each 10 μL reaction consists of 5 μL of master mix, 0.5 μL each of forward and reverse primer (10 μM stock), and 4 μL of diluted sample cDNA. Each qPCR was performed with duplicate samples, and the cycling conditions were 2 min at 50°C, 10 min at 95°C followed by 15 s at 95°C, 45 s at 60°C and 15 s at 95°C for 40 cycles. In addition, a melt curve analysis was performed after amplification to verify product specificity. The relative abundance of the target genes was calculated using the 2^−ΔΔCt^ method [30]. The assessed gene from each dietary group was normalized against the reference diet.

**Table 4:** California yellowtail (*Seriola dorsalis*) primer pairs used in this study for qPCR gene expression, primer efficiency (E), and coefficient of determination (R^2^). These selected primers were previously reported in Viana et al. [27].

	Forward Sequence	Reverse Sequence	E (%)	R^2^
*actb*	TGCGTGACATCAAGGAGAAG	AGGAAGGAAGGCTGGAAGAG	96.8	0.99
*il-1b*	AGCTCTGACAGCGATCTGGT	CCGGATGTTGAAGGTTCTGT	103.1	0.99
*apn*	GTGGAATGACTTGTGGCTCA	ATGTGCTCTGGCTTCAGGAT	109.1	0.99
*mga*	AGCCGACTCCAGCTTTAACA	GACAGAGCTGGAACCCAAGA	97.4	1
*atpase*	TACCGGTGTGGAAGAAGGTC	TTCATAAGCCAGGGAGATGG	110.0	0.99
*gpx1*	TTTACGACCTGAAGGCCAAC	CTCCTGATGTCCGAACTGGT	99.4	1

*actb* (beta-actin), *il-1b* (interleukin-1β), *apn* (aminopeptidase N), *mga* (maltase-glucoamylase), *atpase* (sodium/potassium transporter), *gpx1* (glutathione peroxidase -1)

### Statistical analyses

All data were expressed as means ± standard error of the mean (SEM) and then analyzed using GraphPad Prism 9 (Boston, MA, USA) statistical software. Growth performances were analyzed using a one-way analysis of variance (ANOVA). The normality of the model residuals was assessed using a Shapiro-Wilk test, and variance homogeneity was evaluated using Bartlett’s test. Similar tests were run to compare the proximate composition and gene expression differences between the treatments. A Kruskal–Wallis test was performed to determine differences in histological rankings among the dietary treatments. Mean values were considered significantly different if the *P*-value was less than 0.05.

## Results

### Growth performance, feed utilization, and whole fish analysis

The biological responses of the CYT are summarized in Table 5. Under the reported conditions, dietary treatment did not affect survival (*P* = 0.416), and survival was 100% in all but one treatment group (BD50; 96.7%). Similarly, final body weight (*P* = 0.815), percent weight gain (WG; *P* = 0.243), protein efficiency ratio (PER; *P* = 0.085), and feed conversion ratio (FCR; *P* = 0.815) were not significantly different among dietary groups. Percent weight gain ranged from 705% to 755% and FCR from 1.11 to 1.16, indicating good performance under the reported conditions.

**Table 5:** Final weight, percent weight gain, feed conversion ratio (FCR), and protein retention efficiency (PRE) for California yellowtail (*Seriola dorsalis*) after 60 days of feeding. Basal = 45% commercial SBM control diet; BD50 = 45% commercial SBM + enzyme-treated SBM diet; BD100 = 45% enzyme-treated SBM only diet; HP50 = 45% commercial SBM + fermented SBM diet; HP100 = 45% fermented SBM only diet; Reference = 45% fishmeal control. All data presented are the mean ± SEM (SEM = Standard error of the mean for all) of three replicate tanks.

Treatment diet	Final weight (g)	Percent weight gain (%)	FCR	PRE (%)	Survival (%)
Basal	135.4 ± 3.6	722.6 ± 21.7	1.16 ± 0.01	33.6 ± 0.4	100
BD50	141.3 ± 2.6	755.6 ± 16.1	1.13 ± 0.02	33.4 ± 0.5	96.7±6.7
BD100	137.6 ± 1.9	731.2 ± 10.1	1.16 ± 0.06	35.3 ± 0.6	100
HP50	139.0 ± 3.0	742.4 ± 18.1	1.14 ± 0.03	34.1 ± 0.6	100
HP100	133.5 ± 1.5	705.8 ± 9.3	1.13 ± 0.02	35.0 ± 0.7	100
Reference	141.1 ± 2.3	753.4 ± 15.2	1.11 ± 0.02	36.4 ± 0.9	100
*P*-value	0.246	0.243	0.815	0.085	0.416

The whole body proximate and mineral composition at the end of the trial is presented in Table 6. All examined whole-body nutrients and minerals were statistically similar among dietary groups (*P*>0.05). Whole body protein ranged from 19.2% to 20.3%. Average body lipid ranged from 4.7% to 5.6%. Whole body moisture (71.3% to 72.7%) and whole ash content ranged from 2.7% to 3.4%. sulfur (0.23% to 0.24%), phosphorus (0.53 & to 0.62%), potassium (0.04 to 0.36%), magnesium (0.04%), calcium (0.55% to 0.75%), sodium (0.12 to 0.13%), iron (13.97 to 16.15 ppm), manganese (0.55 to 1.10 ppm), copper (0 to 0.50 ppm), and zinc (12.45 to 14.30 ppm).

**Table 6:** Proximate composition (as is, g 100^−^1) and mineral composition of whole fish after the 8-week feeding trial. Basal = 45% commercial SBM control diet; BD50 = 45% commercial SBM + enzyme-treated SBM diet; BD100 = 45% enzyme-treated SBM only diet; HP50 = 45% commercial SBM + fermented SBM diet; HP100 = 45% fermented SBM only diet; Reference = 45% fishmeal control.

	Initial	Basal	BD50	BD100	HP50	HP100	Reference
Moisture (%)	76.6	72.7 ± 0.4	71.3 ± 0.4	71.8 ± 0.4	72.3 ± 0.4	72.7 ± 0.2	72.7 ± 0.6
Dry (%)	23.4	27.3 ± 0.4	28.7 ± 0.4	28.2 ± 0.4	27.7 ± 0.4	27.3 ± 0.2	27.3 ± 0.6
Protein (%)	15.2	19.7 ± 0.4	19.4 ± 0.3	19.5 ± 0.1	20.3 ± 0.2	19.2 ± 0.2	19.7 ± 0.4
Fat (%)	3.48	4.8 ± 0.2	4.7 ± 0.6	5.1 ± 0.3	5.6 ± 0.4	4.9 ± 0.3	5.0 ± 0.5
Ash (%)	3.15	2.9 ± 0.4	3.4 ± 0.3	3.1 ± 0.1	2.9 ± 0.4	2.7 ± 0.2	2.8 ± 0.3
Sulfur (%)	0.24	0.23± 0.00	0.23 ± 0.01	0.23 ± 0.00	0.23 ± 0.00	0.23 ± 0.00	0.24 ± 0.00
Phosphorus (%)	0.58	0.57 ± 0.02	0.62 ± 0.06	0.57 ± 0.02	0.54 ± 0.02	0.57 ± 0.02	0.53 ± 0.03
PHRE (%) ^1^		0.29 ± 0.01	0.33 ± 0.04	0.32 ± 0.02	0.26 ± 0.01	0.32 ± 0.01	0.30 ± 0.02
Potassium (%)	0.36	0.35 ± 0.01	0.35 ± 0.00	0.35 ± 0.00	0.35 ± 0.01	0.35 ± 0.01	0.36 ± 0.00
Magnesium (%)	0.05	0.04 ± 0.00	0.04 ± 0.00	0.04 ± 0.00	0.04 ± 0.00	0.04 ± 0.00	0.04 ± 0.00
Calcium (%)	0.8	0.63 ± 0.04	0.75 ± 0.11	0.64 ± 0.03	0.58 ± 0.04	0.64 ± 0.05	0.55 ± 0.06
Sodium (%)	0.25	0.13 ± 0.01	0.13 ± 0.01	0.12 ± 0.01	0.13 ± 0.01	0.13 ± 0.01	0.13 ± 0.01
Iron (ppm)	13.4	13.97 ± 0.37	14.05 ± 0.28	14.07 ± 0.40	14.05 ± 0.38	15.97 ± 1.15	16.15 ± 1.18
Manganese (ppm)	1.6	0.95 ± 0.34	1.05 ± 0.36	1.10 ± 0.04	0.55 ± 0.32	0.88 ± 0.30	0.60 ± 0.35
Copper (ppm)	0	0.50 ± 0.29	0 ± 0.00	1 ± 0.00	0.25 ± 0.25	0.25 ± 0.25	0.25 ± 0.25
Zinc (ppm)	16.4	12.45 ± 0.52	12.88 ± 0.65	13.30 ± 0.64	14.30 ± 0.34	13.45 ± 0.13	12.60 ± 0.93

^1^ Phosphorus retention efficiency: [(final total body phosphorus − initial total body phosphorus)/total dietary phosphorus intake] × 100

### Gut histology, immune and physiological gene expression

**Fig 1:**
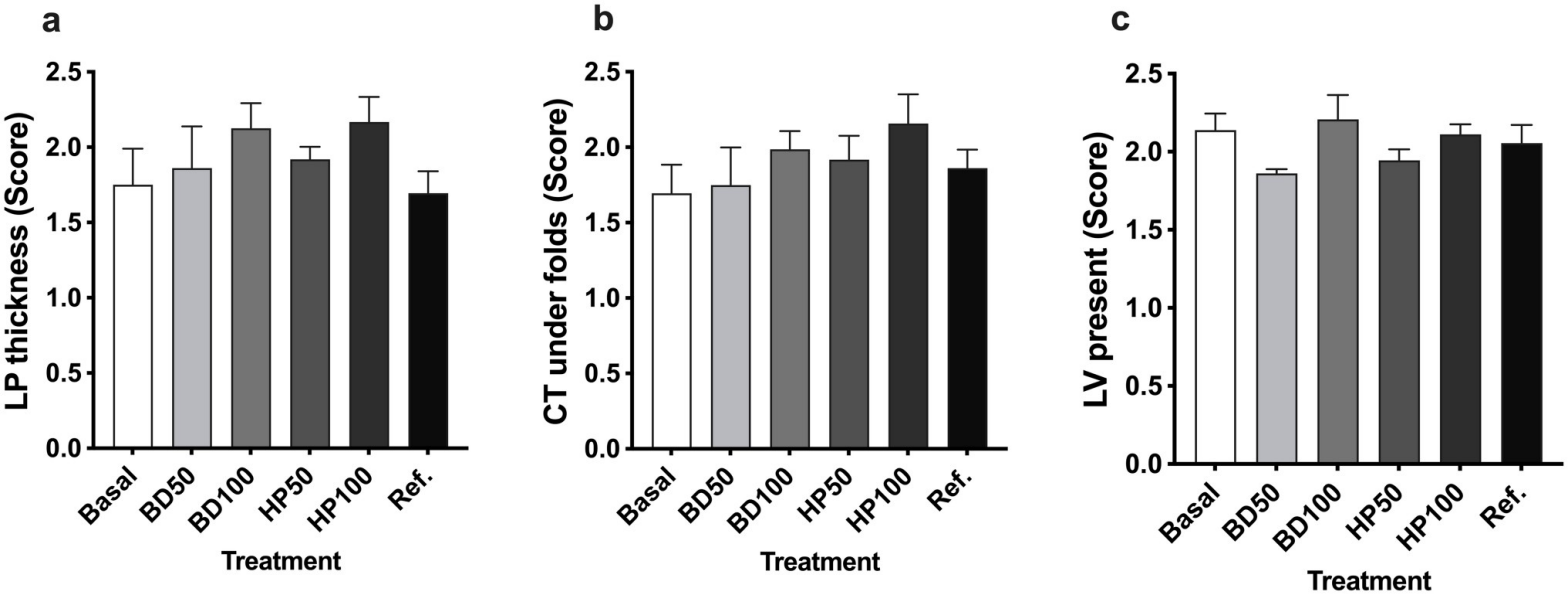
Qualitative histological assessment of *CYT* distal intestine slides after dietary treatment. **a)** indicates the lamina propria (LP) thickness; **b)** the amount of connective tissues (CT); and **c)** the presence of large vacuoles (LV). Values are depicted as mean and SEM (standard error of the mean for all). Basal = solvent extracted soybean-based diet; BD50 = 50% Bright Day soybean-based diet; BD100 = 100% Bright Day soybean-based diet; HP50 = 50% HP300 soybean-based diet; HP100 = 100% HP300 soybean-based diet and Ref. = fishmeal reference diet.

The intestinal qualitative measurements after eight weeks of dietary feeding are summarized in Fig 1. There were no statistical differences in morphology scores for lamina propria thickness (*P* = 0.256), the amount of connective tissue (*P* = 0.618), and number of large vacuoles present (*P* = 0.158) among the dietary treatments. This indicated that the partial replacement of fishmeal with the improved soybean products or when combined with commercial SE soybean meal in the diet did not affect the intestinal morphology of CYT.

**Fig 2:**
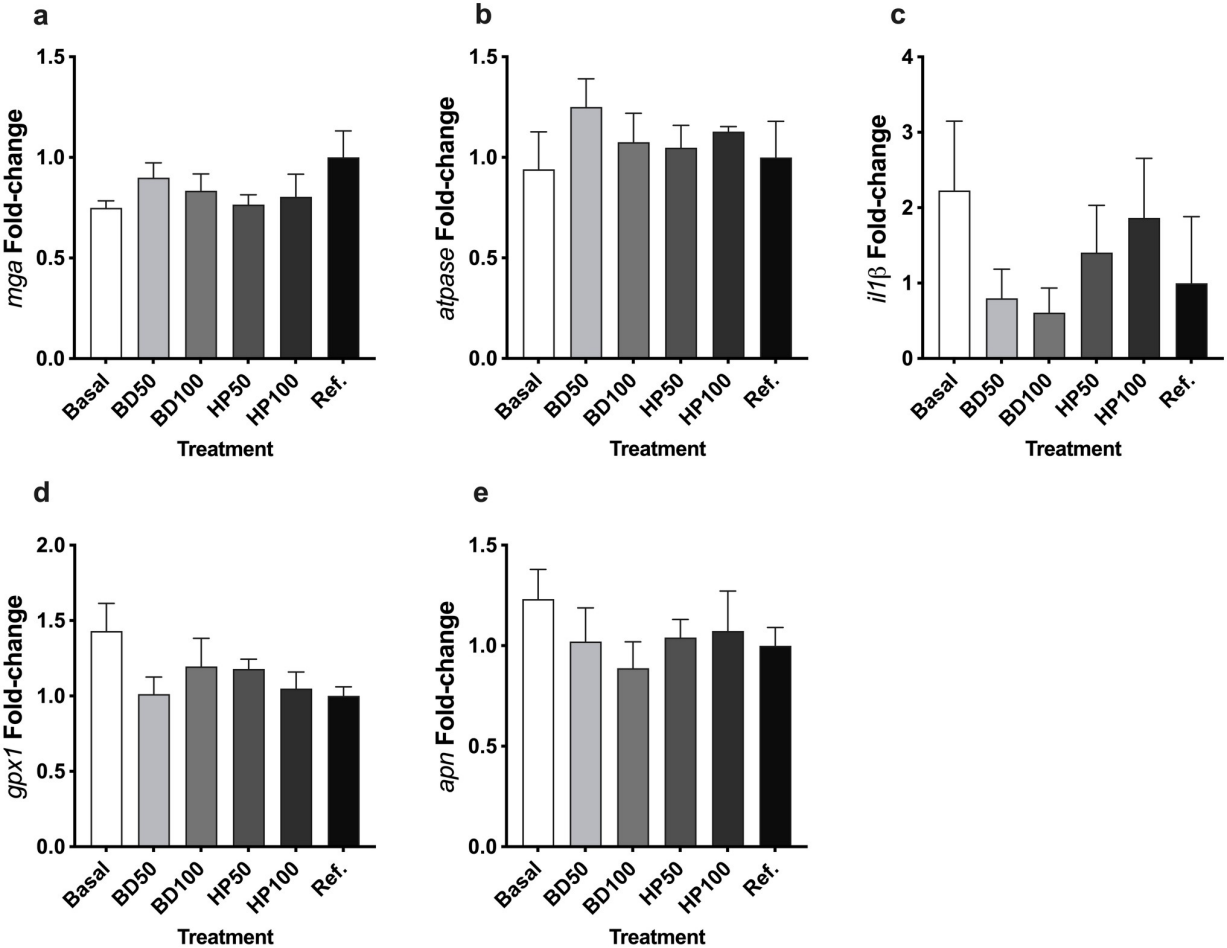
**a)** *mga* expression (fold-change), **b)** *atpase* expression (fold-change), **c)** *il-ib* expression (fold-change), **d)** *gpx1* expression (fold-change), and **e)** *apn* expression (fold-change) was evaluated from the extracted gut of CYT. All data presented are the mean ± SEM of three replicate tanks. Basal = solvent extracted soybean-based diet; BD50 = 50% Bright Day soybean-based diet; BD100 = 100% Bright Day soybean-based diet; HP50 = 50% HP300 soybean-based diet; HP100 = 100% HP300 soybean-based diet and Ref. = fishmeal reference diet. Gene expression data were first normalized to *actb* and then adjusted to the reference dietary treatment group for comparison (2^−ΔΔCt^ methodology).

Immune, physiological, and digestion-related cytokines were quantified in the guts of fish fed all six diet treatments (Fig 2). However, no significant dietary difference was found in the fold-change of *mga* (*P* = 0.397), *atpase* (*P* = 0.590), *apn* (*P* = 0.687), *il-1β* (*P* = 0.659), and *gpx-1* (*P* = 0.279).

## Discussion

Protein derived from soybean has become a critical component of aquaculture diets and is currently one of the primary protein sources in most feed formulations. This is because of its stable nutrient profile, worldwide availability, and cost. Although it offers a cheaper alternative to most animal-based proteins, the anti-nutritional factors (ANFs) present in soybean could lead to intestinal inflammation and damaged immune response in aquatic species [31, 32] and at high dietary inclusion levels it, may decrease the growth of the culture species [33, 34].

Previous studies have reported the successful substitution of fishmeal with SBM in the diet of CYT [2]. However, further investigation into intestinal integrity revealed that an inclusion level above 10% might expose CYT to enteritis [27, 35]. Therefore, this study evaluated the growth, intestinal characteristics, immune, and physiological gene expression resulting from including improved SBM in the diet of CYT.

Under the conditions of this study, growth performance was good, and tissue replacement was high, with percent weight gain ranging from 705–755%, yet there were no significant differences between dietary treatments including food conversion and protein efficiency ratios. Thus, the processed SBMs used in the study and the different inclusion levels did not cause a differential growth and were similar to a reference diet that did not contain soy-based proteins. Fish survival was very good among all treatments at >96% and was not found to be statistically different. Jirsa et al. [2] noted that the growth performance of CYT decreased with increasing substitution of fishmeal with SBM. The authors inferred that the increasing level of SBM contributed to increasing nutritional deficiency or palatability issues caused by increased inclusion levels, which also agrees with previous studies in other fish species [36, 37]. However, the use of improved SBM products has been previously suggested to safely constitute certain inclusion levels, more than commercial solvent-extracted SBM, in several fish species, including rainbow trout (*Oncorhynchus mykiss*) [19, 38], largemouth bass (*Micropterus salmoides*) [36], and hybrid striped bass (*Morone saxatilis* x *M*. *chrysops*) [39] without reduction in growth performance.

The body proximate composition of fish and the amino acid content within muscle tissue significantly influence the nutritional value of fish fillets, which is a key consideration for consumers [40]. In marine fish aquaculture, previous research has underscored the influence of dietary composition, especially high inclusion levels of SBM, in enhancing moisture content while reducing the levels of crude protein and lipids within the body composition of various fish species, including 50% FM replacement in Japanese seabass (*Lateolabrax japonicus*) [41], 80% FM replacement in giant grouper (*Epinephelus lanceolatus*) [42], and 60% FM replacement in spotted rose snapper (*Lutjanus guttatus*) [43]. Their results suggest that body nutrients increased with reduced SBM inclusion levels. In the case of the California yellowtail examined in this study, 20% FM was replaced by SBM variants, resulting in similar body compositions. Supporting this, Zhou et al. [44] found no discernible differences in the body nutrient composition of cobia (*Rachycentron canadum*) when up to 30% of FM was substituted with SBM. Nevertheless, it is noteworthy that the utilization of enhanced SBM products has contributed to improvements in the body composition of fish. For example, including soybean protein concentrates and SBM led to increased body fat content in white snook (*Centropomus viridis*) [45]. Similarly, in a related investigation, Jae et al. [46] suggested a potential correlation between processed SBM and the PPAR-γ gene, which plays a pivotal role in regulating fatty acid synthesis.

Despite no apparent differences in overall growth and body nutrients in our study, there could be morphological changes in the gut since previous studies have reported changes in the intestine structure when SBM was included in the diet of CYT [27] and yellowtail kingfish (*S*. *lalandi*) [47]. Comparatively, fermented SBM showed less adverse effects on the intestinal structure of largemouth bass [36], Florida pompano [48], and turbot (*Scophthalmus maximus*) [49]. Although processed SBM may reduce the ANF contents of SBM through degradation by processing [36, 50], which would allow for an increase in SBM inclusion levels in fish diet. Concerning the inclusion levels, Shiu et al. [51] reported that more than 30% of FM replacement by fermented soybean meal (FSBM) produced pathomorphological changes in orange-spotted grouper (*Epinephelus coioides*, Hamilton), and more than 40% of FM replacement by FSBM decreased the villus height in rainbow trout [52]. However, the increase in lamina propria thickness and the number of connective tissues were significantly greater in rainbow trout fed 50% FSBM [19]. The lack of gut inflammation in this study suggests that the diets, regardless of the SBM product used in this study and varying levels, did not elicit evident antigenic stimulation that may cause gastrointestinal tract (GIT) complications. In a similar experiment by Bruce et al. [38], no changes in the intestinal characteristics of rainbow trout or apparent enteritis resulted from different processed soy diets. In contrast, Novriadi et al. [48] found lower cell infiltration of the submucosa and lamina propria in Florida pompano fed 75% and 100% FSBM diets. While we did not find such differences in any of the SBM levels included, it can be inferred that the discrepancies are related to the high inclusion level and domestication across species, which might contribute to the tolerance of dietary variation [53]. Thus, based on these findings, replacing SBM with processed soy may serve as a promising ingredient to partly prevent various physiological abnormalities that may occur in the distal intestine of CYT with a plant-based diet. Further studies should investigate the long-term replacement of FM with increased inclusion levels of the processed SBMs used in this study and the effects on intestinal morphology.

The relevance of gut health in finfish aquaculture has increased in recent years due to the emergence of various gastrointestinal disorders that have hindered the development of the industry [54]. The SBM-induced condition of “enteritis” has been directly correlated with poor expression patterns of digestive enzymes and inflammatory genes in yellowtail kingfish (*S*. *lalandi*) [47] and California yellowtail (*S*. *dorsalis*) [27], alongside histological assessment. In *S*. *lalandi*, previous analysis of intestinal histology did not reveal any major signs of enteritis when the fish were fed a SPC [55] or solvent-extracted SBM diets [56]. The same studies reported changes in aspects of digestive enzymes, thus using additional assays and tools for assessing signs of inflammation or stress due to diet is useful in assessing diets., In *S*. *dorsalis*, Viana et al. [27] reported cytokines expression profiles in the guts after varied SBM inclusion levels, which were fundamental to oxidative stress and antioxidant enzymes. The study reported a specific expression pattern that followed an increase in SBM inclusion level, an inflammatory indicator (*il1b*), protein digestive enzyme (*apn*), and disaccharides digestive enzyme (*mga*), which illustrated a significant immune compromise after 30 days feeding trial. Their results were also supported by differences observed in the intestinal morphology. To better understand the effect of Bright Day and HP300 soybean and inclusion levels on gut health, we investigated the gene expression of key health markers as previously published by Viana et al. [27]. Contrary to their findings, none of the quantified genes were significantly expressed among all treatments in our study. However, the lack of differential gut histological morphology in our study may corroborate the similarities observed in the genes expressed in the guts. Dam et al. [57] conducted a transcriptomic analysis of intestinal tissues of *S*. *lalandi* and found that faba bean meal was a good potential plant-based protein source for this species. Similar molecular evaluations have also been conducted using shrimp, where Xie et al. [32] saw affected antioxidant enzyme activity, including catalase (*cat*) and superoxide dismutase (*sod*) in the hemolymph after the FM level decreased to 15% having been replaced with SBM and SPC.

In conclusion, this study demonstrated the potential use of bioprocessed SBM products and replacement of commercial SBM in the diets of CYT, illustrated by growth performance, intestinal morphology, and gene expression of immune, physiological, and antioxidant enzymes, which was indifferent in all the dietary treatments, including the fishmeal diet. Although previous works have reported the inclusion of SBM and limitations due to “enteritis” in CYT, the present results suggest that advanced SBMs could limit FM inclusion to 10% while replacing up to 100% of commercial SBM without compromising intestinal integrity. These findings bode well for commercial CYT production and future investigation to further refine the inclusion of SBM in the diets.

## Supporting information

S1 Fig Histomicrographs of distal intestines from California yellowtail after 60 d of dietary treatment. **(a)** Basal diet with 100% solvent-extracted soybean as SBM source, **(b)** Diet with 50% bright day SBM variant (BD50), **(c)** Diet with 100% bright day SBM variant (BD100), (d) Diet with 50% Hamlet SBM variant (HP50), **(e)** Diet with 100% Hamlet SBM variant (HP100) or **(f)** Soybean meal-free reference diet. Note the yellow arrow indicates the connective tissues at the base of the folds, the green arrow indicates the lamina propria and the red arrow indicates large vacuoles.
(TIF)

S1 Table Growth performance table. (XLSX)

S2 Table Gut histology grades table. (XLSX)

S3 Table Tables showing gene expression data. (XLSX)
