# Supplementary figures and images for: Practical diets for California yellowtail, Seriola dorsalis: Use of advanced soybean meal products on growth performance, body composition, intestinal morphology, and immune gene expression
Source: PLoS One. 2024 Jun 7;19(6):e0304679. doi: 10.1371/journal.pone.0304679 (PMC11161020; doi:10.1371/journal.pone.0304679)

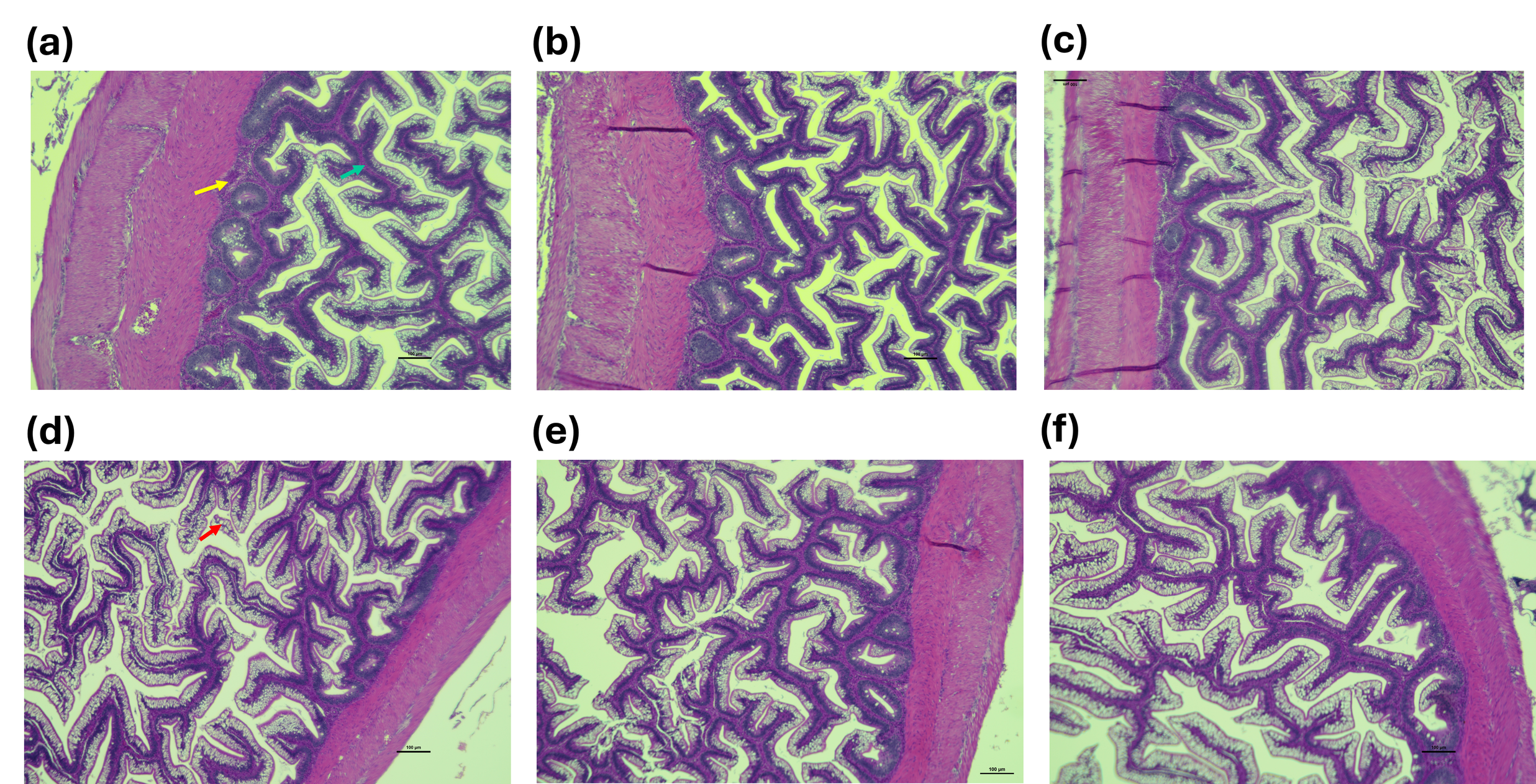

Supplement: S1 Fig — (a) Basal diet with 100% solvent-extracted soybean as SBM source, (b) Diet with 50% bright day SBM variant (BD50), (c) Diet with 100% bright day SBM variant (BD100), (d) Diet with 50% Hamlet SBM variant (HP50), (e) Diet with 100% Hamlet SBM variant (HP100) or (f) Soybean meal-free reference diet. Note the yellow arrow indicates the connective tissues at the base of the folds, the green arrow indicates the lamina propria and the red arrow indicates large vacuoles. (TIF) [file pone.0304679.s001.tif]
